# Tanshinone I inhibited growth of human chronic myeloid leukemia cells via JNK/ERK mediated apoptotic pathways

**DOI:** 10.1590/1414-431X2020e10685

**Published:** 2021-05-24

**Authors:** Siya Sun, Lingyan Zhu, Mengru Lai, Rubin Cheng, Yuqing Ge

**Affiliations:** 1 The First Affiliated Hospital of Zhejiang Chinese Medical University, Hangzhou, China; 2 College of Pharmaceutical Science, Zhejiang Chinese Medical University, Hangzhou, China

**Keywords:** Tanshinone I, Chronic myeloid leukemia, K562, Apoptosis, JNK/ERK signaling pathway

## Abstract

Tanshinone I (Tan I) is one of the main bioactive ingredients derived from *Salvia miltiorrhiza* Bunge, which has exhibited antitumor activities toward various human cancer cells. However, its effects and underlying mechanisms on human chronic myeloid leukemia (CML) cells still require further investigation. This study determined the effects and mechanisms of anti-proliferative and apoptosis induction activity induced by Tan I against K562 cells. The cytotoxic effect of Tan I at varying concentrations on K562 cells was evaluated via MTT assay. Cell apoptosis was further investigated through DAPI staining and flow cytometry analysis. The expression levels of apoptosis-related proteins and activities of JNK/ATF2 and ERK signaling pathways were analyzed by western blot. Quantitative PCR was performed to further determine mRNA expression levels of JNK1/2 and ERK1/2 after Tan I treatment. The results indicated that Tan I significantly inhibited K562 cell growth and induced apoptosis in a concentration- and time-dependent manner. It induced significant cellular morphological changes and increased apoptosis rates in CML cells. Tan I promoted the cleavages of caspase-related proteins, as well as increased the expression levels of PUMA. Furthermore, Tan I significantly activated JNK and inhibited ATF-2 and ERK signaling pathways. The mRNA expression levels of JNK1/2 and ERK1/2 were up-regulated by Tan I, further confirming its regulatory effects on JNK/ERK signaling pathways. Overall, our results indicated that Tan I suppressed cell viability via JNK- and ERK-mediated apoptotic pathways in K562 cells, suggesting that it might be a promising candidate as a novel anti-leukemia drug.

## Introduction

Chronic myeloid leukemia (CML) is a myeloproliferative disorder characterized by the genetic translocation between chromosomes 9 and 22, which gives rise to the Philadelphia (Ph) chromosome and leads to the generation of the BCR-ABL oncogenic fusion gene (1,2). The BCR-ABL oncogene encodes the Bcr-Abl protein with constitutive kinase activity. According to a previous study, 2.4% of all new cancer cases and 3.2% of cancer deaths were attributed to leukemia in 2018 (3). The tyrosine kinase inhibitor (TKI) imatinib has become the first-line treatment for the chronic-phase of CML patients, which markedly improved their quality of life. However, up to 40% of patients show substantial resistance or intolerance to TKIs, which seriously limits the attainment of treatment-free remission or a cure (4). Additionally, treatment with imatinib has been documented to have significant safety issues. Thus, developing new drugs is urgently needed for CML therapy to overcome these problems.

Mitogen-activated protein kinases (MAPKs) are able to sense the changes in cellular conditions. Major mammalian MAPK subfamilies include c-Jun N terminal kinase (JNK), p38 MAPK, and extracellular signal-regulated kinases 1 and 2 (ERK1/2), which are not only related to pro-apoptotic action but also to anti-apoptotic action in the cell (5). The JNK signaling cascade is involved in numerous cellular stresses where it regulates a wide range of cellular processes including cell proliferation, apoptosis, differentiation, inflammation, and others (6). Mc178-Ab induced A549 cells apoptosis via activating JNK and p38 pathway, while the phosphorylation of ERK and Akt was decreased (7). Daucosterol blocked prostate cancer growth by increasing the phosphorylation of JNK and leading to autophagic dependent apoptosis, where JNK-specific inhibitor SP600125 could abate the daucosterol-mediated autophagy and apoptotic cell death (8). In addition, the natural active monomer EM-2 promoted G2/M phase arrest and apoptosis by activating the JNK pathway in hepatocellular carcinoma cells, which could be partially reversed when treated with the JNK inhibitor SP600125 (9). The pro-apoptotic JNK signaling pathway was therefore considered a potential target for cancer therapy, which has been documented as one of the major apoptosis mediators for CML cells (10). Furthermore, Tanshinone IIA exhibited mitochondria-dependent apoptosis induction by increasing the phosphorylation of JNK protein in KBM5 cells (11). Thus, identifying bioactive agents targeting JNK pathway is a promising approach for the development of anti-leukemia drugs.

Recent studies have demonstrated that *Salvia miltiorrhiza* Bunge as well as its abietane diterpene isolates exhibited therapeutic potentials targeting several malignant tumors. Tanshinone I (Tan I) is a major lipid-soluble compound extracted from *S. miltiorrhiza*, which has exhibited significant antitumor activities toward a variety of human cancer cells (12). Specifically, Tan I inhibits proliferation and metastasis and induces apoptosis via the suppression of JAK/STAT3 signaling pathway in osteosarcoma (13). Furthermore, Tan I recovers cisplatin sensitivity of cervical cancer in a KRAS-dependent manner (14). Although Tan I has shown promising application prospects in cancer treatment, emerging evidence suggests that it functions in a cell-type-specific manner to affect roles of regulatory pathways that modulate tumor proliferation, apoptosis, and cell migration (15). The anti-leukemia effects of Tan I on human CML cells and its specific underlying mechanisms still require further investigation.

In this study, we reported the anti-leukemia effect of Tan I on the cell viability and apoptosis of K562 cells and investigated its underlying mechanisms.

## Material and Methods

### Reagents

**Figure 1:**
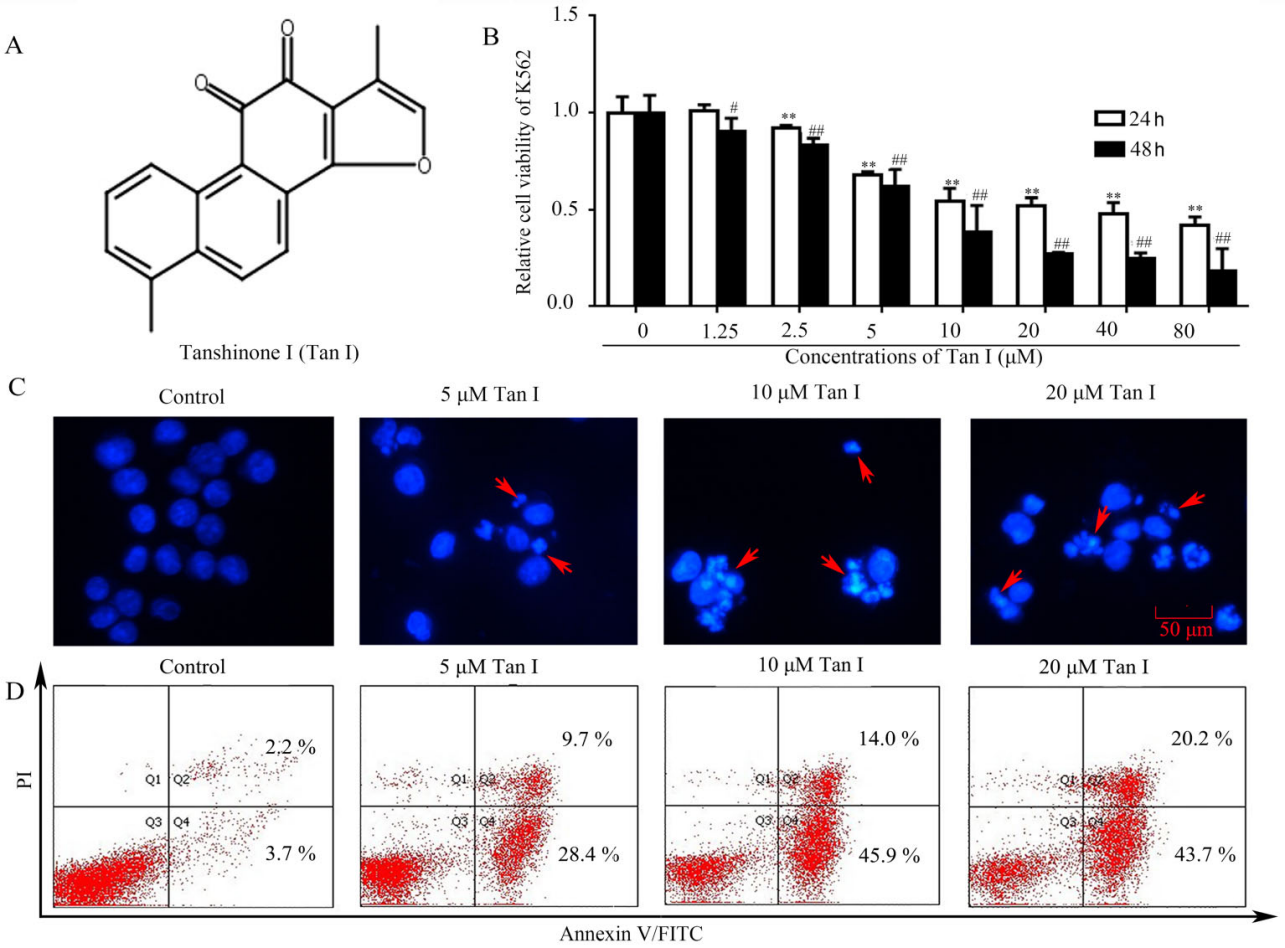
Tanshinone I treatment inhibited growth and induced apoptosis in human chronic myeloid leukemia K562 cells. **A**, The chemical structure of Tan I. **B**, Effect of Tan I concentration on K562 cell viability, comparing 24 and 48 h of exposure. **C**, Bright nuclear condensation and perinuclear apoptotic bodies were captured by fluorescent microscopy (scale bar 50 μm). **D**, Apoptosis rate of K562 after the treatment of different concentrations of Tan I. Data are reported as means±SD. **P<0.01 compared with the control group at 24 h; ^#^P<0.05 and ^##^P<0.01 compared with the control group at 48 h (ANOVA).

Tan I (purity ≥98%) was purchased from Yuanye Bio-Technology (China) and its structure is shown in Figure 1A. The drug was dissolved in DMSO and stored at -20°C. RPMI 1640 medium was obtained from Cellmax (China) and the fetal bovine serum (FBS) was from Sijiqing (China). MTT assay kit and DAPI-staining kit were obtained from Beyotime Biotechnology (China) and Annexin V-FITC+PI double-staining kit was purchased from BD Biosciences (USA). RNA-Quick Purification Kit was purchased from Yishan Biotechnology (China) and both the mRNA reverse transcription kit and RT-PCR kit were obtained from Yeasen Biotechnology (China). BCA Protein Assay Kit was from Beyotime Biotechnology and the antibodies used in this study were purchased from Cell Signaling Technology (USA): cleaved caspase 3 (Cat 9664), cleaved caspase 9 (Cat 9505), cleaved PARP (Cat 5625), PUMA (Cat 12450P), Bcl-2 (Cat 3498), phospho-SAPK/JNK (Thr183/Tyr185) (Cat 4668), JNK (Cat 9252), phospho-p44/42 MAPK (Erk1/2) (Thr202/Tyr204) (Cat 4370), phospho-ATF-2 (Thr71) (Cat 5112), and β-actin (Cat 3700). Additionally, ERK1/2 (Cat AF0155) was from Affinity Biosciences (USA) and secondary antibodies were obtained from Santa Cruz Biotechnology (USA).

### Cell lines and culture

K562 cell line was purchased from the Cell Bank of Type Culture Collection of Chinese Academy of Sciences (China). Cells were maintained in RPMI-1640 medium containing 10% of FBS and incubated in a humidified atmosphere with 5% CO_2_ at 37°C.

### MTT assay

Cell viability was tested by MTT assay, according to the manufacturer's instructions. Briefly, 1×10^4^ cells were incubated with different concentrations of Tan I (0, 1.25, 2.5, 5, 10, 20, 40, and 80 µM) for 24 and 48 h. Then, 10 µL MTT (5 mg/mL) solution was added into each well. After 4 h, 150 µL DMSO was added to dissolve the formazan. The absorbance was detected at 490 nm using a microplate reader (Thermo Fisher Scientific, Finland). The cell viability of 0 µM served as the control group for 24 and 48 h.

### DAPI staining

The major characteristics of apoptosis (DNA condensation and fragmentation) were evaluated by DAPI staining. K562 cells (5×10^5^) were seeded onto six-well plates at a series of concentrations of Tan I (0, 1.25, 2.5, 5, 10, and 20 µM) for 48 h. Then, cells were fixed with 4% paraformaldehyde in the dark for 20 min. Next, cells were washed with ice-cold PBS twice before incubated with 0.5 µg/mL DAPI in the dark for 20 min at room temperature. Apoptotic nuclei were observed under 200× magnification using an IX-71 inverted fluorescence microscope (Olympus, Japan). Cells treated with 0 µM Tan I were used as the control group.

### Flow cytometry analysis

Flow cytometry with Annexin-V-FITC/PI apoptosis kit was used to detect cell apoptosis. First of all, 5×10^5^ cells/well were seeded onto six-well plates and incubated with a series of concentrations of Tan I (0, 1.25, 2.5, 5, 10, and 20 µM) for 48 h. K562 cells were collected and washed with ice-cold PBS twice. Then, 1×10^6^ cells were re-suspended in 100 µL binding buffer containing 5 µL Annexin V-FITC conjugate and 5 µL PI. The cells were incubated in the dark for 20 min at room temperature. After adding 300 µL of 1× Annexin V-FITC binding buffer, 1×10^4^cells were collected and analyzed by the FACScalibur flow cytometer (Becton Dickinson, USA). Cells treated with 0 µM Tan I were used as the control group.

### Quantitative real-time PCR

K562 cells were seeded onto six-well plates at 5×10^5^ per well and incubated with different concentrations of Tan I (0, 1.25, 2.5, 5, 10, and 20 µM) for 48 h or 5 µM for different times (0, 0.5, 1, 2, 4, and 6 h). Total RNA was extracted from K562 cells using RNA-Quick Purification Kit and reverse transcribed to complementary DNA (cDNA) using the 1st Strand cDNA Synthesis SuperMix Kit (Yeasen Biotechnology, China). For the reactions, SYBR green PCR master mix and 7900 HT FAST Real-Time PCR instrument (Applied Biosystems, USA) were applied. PCR thermocycling conditions were 40 amplification cycles, denaturation step at 95°C for 15 min, followed by 40 cycles at 95°C for 15 s and 60°C for 30 s. The primer sequences used for GAPDH, JNK1, JNK2, ERK1, ERK2 were as follows: GAPDH, forward primer, 5′-CCCAGAAGACTGTGGATGG-3′, reverse primer, 5′-TTCAGCTCAGGGATGACCTT-3′; JNK1, forward primer, 5′-GACGCCTTATGTAGTGACTCGC-3′, reverse primer, 5′-TCCTGGAAAGAGGATTTTGTGGC-3′; JNK2, forward primer, 5′-TACGTGGTGAC-ACGGTACTACC-3′, reverse primer, 5′-CACAACCTTTCACCAGCTCTCC-3′; ERK1, forward primer, 5′-TGGCAAGCACTACCTGGATCAG-3′, reverse primer, 5′-GCAGAGACTGTAGGTAGTTTCGG-3′; ERK2, 5′-ACACCAACCTCTCGTACATCGG-3′, reverse primer, 5′-TGGCAGTAGGTCTGGTGCTCAA-3′. The relative mRNA expression was assessed using the 2^-ΔΔCt^ method. Cells treated with 0 µM Tan I were used as the control group. All experiments were performed in triplicate.

### Western blot analysis

The western blot technique was applied to analyze the protein expression in K562 cells after treatment with Tan I as previously described (16). Briefly, K562 cells were plated onto six-well plates with a concentration of 5×10^5^ cells/well and treated with different concentrations of Tan I (0, 1.25, 2.5, 5, 10, and 20 µM) for 48 h. Then, K562 cells were harvested and lysed with RIPA lysate buffer with 1 mM PMSF and phosphatase inhibitor for 30 min. The BCA protein concentration determination kit was used to quantify the concentrations of samples. Equal amounts of sample proteins were electrophoretically separated and then transferred to PVDF membranes (Millipore, USA) by PowerPac Basic Supply 164-5050 (Bio-Rad Laboratories, USA). The membranes were blocked with 5% (w/v) nonfat dry milk and 0.1% Tween 20 in tris-buffered saline (TBS) at room temperature for at least 1 h, and then incubated with the target antibodies (cleaved caspase 3/9, cleaved PARP, PUMA, Bcl-2, p-JNK, JNK, p-ERK 1/2, ERK1/2 p-ATF2, and β-actin) overnight at 4°C, respectively. After washing with tris-buffered saline and Tween^®^ 20 (TBST) three times, the membranes were incubated with anti-rabbit secondary antibody or anti-mouse secondary antibody for 1 h at room temperature. The enhanced chemiluminescence reagent (Fude Biological Technology, China) was used to detect the immunoreactive protein bands. Images were captured and analyzed using the Image Lab system (Bio-Rad Laboratories). Cells treated with 0 µM Tan I were used as the control group.

### Statistical analysis

All data are reported as means±SD and one-way analysis of variance (ANOVA) was performed by GraphPad Prism 7.0 software. Significant differences were defined as P<0.05.

## Results

### Tan I reduced cell viability and induced cell apoptosis in K562 cells

As shown in Figure 1B, Tan I exhibited inhibiting effects on viability of K562 cells in a dose- and time-dependent manner. With the treatment of 2.5 µM Tan I for 24 h, the growth inhibitory rate was just 8.02%, while the inhibitory rate reached 32.04% when the concentration of Tan I increased to 5 µM, which was 3-fold higher than that of 2.5 µM. The IC_50_ values of Tan I on K562 cells were 29.62 and 8.81 μM for 24 and 48 h, respectively. To further evaluate the anti-leukemia underlying mechanism of Tan I, cell apoptosis was detected via DAPI staining and flow cytometry analysis on K562 cells. The staining results indicated that Tan I-treated cells presented typical apoptotic features, including bright nuclear condensation and perinuclear apoptotic bodies (Figure 1C). Furthermore, Tan I increased the cell apoptosis percentages in a concentration-dependent manner (Figure 1D). In the control group, the apoptosis percentage of K562 cells was 5.9%, while the percentage of apoptotic cells increased to 38.1 and 59.9% after the treatment of 5 and 10 µM of Tan I, respectively, which was 5.46- and 9.15-fold higher than that of the control group. These results were consistent with cell viability results. Our results suggested Tan I could inhibit cell viability and induce apoptosis in K562 cells in a concentration-dependent manner, providing a potential therapeutic agent for human chronic myeloid leukemia.

### Tan I activated cleavage of caspase proteins and increased protein levels of PUMA

**Figure 2:**
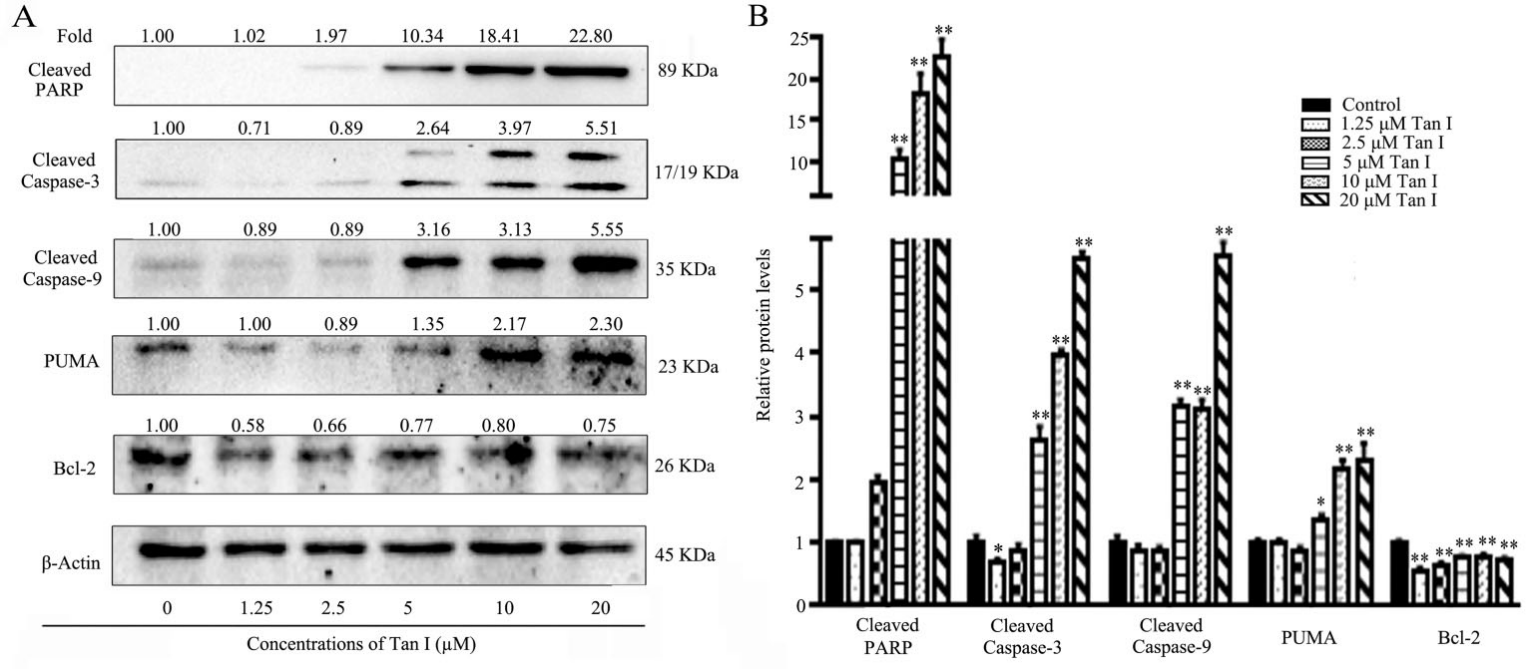
Effects of apoptosis-related protein in K562 cells after the treatment of Tan I. **A**, Protein expression of cleaved caspase 3/9, cleaved PARP, PUMA, and Bcl-2 were analyzed by western blot. **B**, The protein expression levels were quantified by Image Lab software. Data are reported as means±SD. *P<0.05 and **P<0.01 compared with the corresponding control group (ANOVA).

As shown in Figure 2, compared with the control group, the expressions of cleaved PARP, caspase-3, and caspase-9 were significantly increased after the treatment of Tan I. Furthermore, Tan I also significantly enhanced the expression of PUMA, a BH3-only pro-apoptotic protein of Bcl-2 family protein, in a concentration-dependent manner. The expression levels of cleaved PARP increased by 0.97-fold after the treatment of 2.5 µM Tan I in K562 cells (Figure 2A). When the concentration of Tan I increased to 5 and 10 µM, the expression level of activated PUMA was 9.34- and 17.41-fold higher than that of the control group, respectively. In addition, Tan I treatment exhibited a stronger up-regulation effect on the expression of PARP than that of caspase-3 and caspase-9 (Figure 2A and B). The activities of cleaved caspase-3, -9, and PARP were increased by 1.64-, 2.16-, and 9.34-fold after the exposure of 5 µM Tan I, suggesting PARP was more sensitive toward Tan I in CML cells. Furthermore, high concentrations of Tan I also significantly increased the protein level of pro-apoptosis mediator PUMA in CML cells. The treatment with 5 and 10 µM Tan I increased the production of PUMA by 0.35- and 1.17-fold compared to that of the control group. However, Tan I showed a slight regulatory effect on anti-apoptosis Bcl-2 protein, indicating an apoptosis regulatory mechanism independent of Bcl-2 inhibition. These results demonstrated that Tan I triggered the apoptosis of K562 cells through the activation of PARP and caspase-3 and -9 proteins *in vitro*.

### Tan I induced the activation of JNK and blocked the activity of ATF-2 and ERK pathways

**Figure 3:**
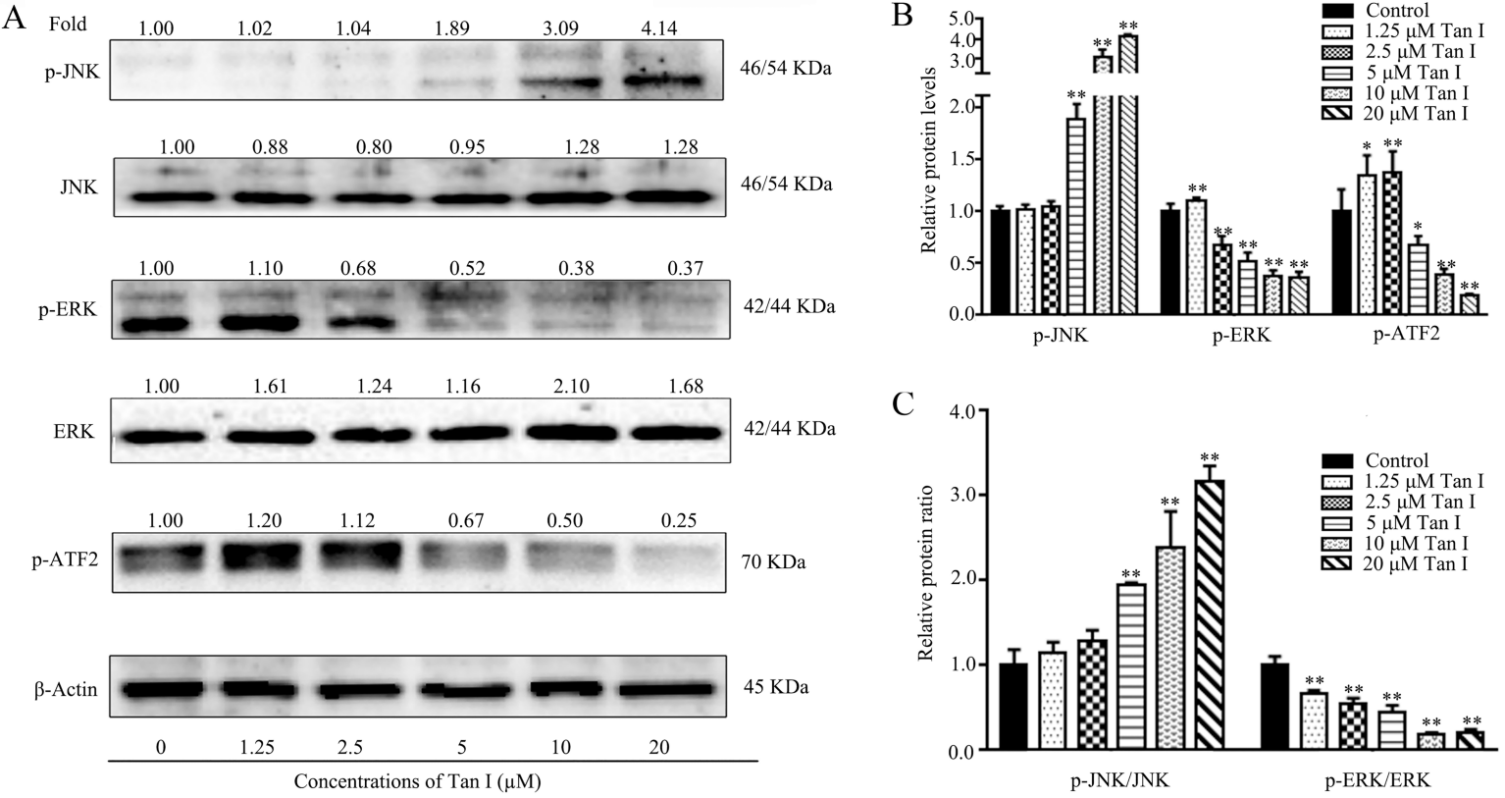
Effect of Tan I on the activities of JNK and ERK signaling pathways at different concentrations. **A**, Related protein expression levels of Tan I in K562 cells were determined with specific antibodies by western blotting. **B**, The phosphorylation levels of JNK, ERK, and ATF2 were quantified by Image Lab software. **C**, The relative protein level ratios of p-JNK/JNK and p-ERK/ERK. Data are reported as means±SD. *P<0.05 and **P<0.01 compared with the corresponding control group (ANOVA).

**Figure 4:**
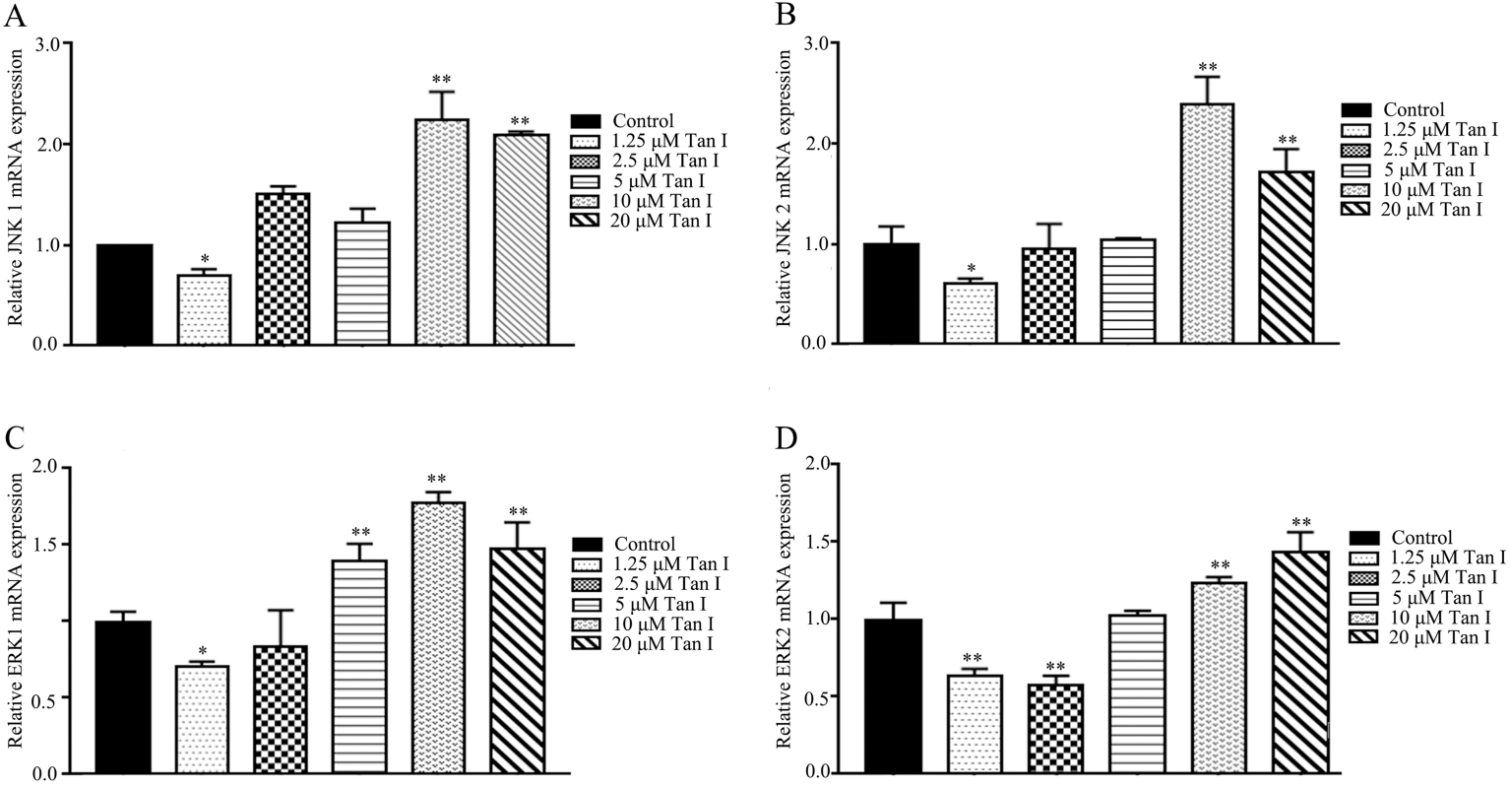
A-**D**, The relative mRNA levels of JNK1/2 and ERK1/2 were evaluated by quantitative PCR analysis. Data are reported as means±SD. *P<0.05 and **P<0.01 compared with the corresponding control group (ANOVA).

As shown in Figure 3, Tan I increased the phosphorylation level of JNK, as well as decreased the expressions of p-ATF2 and p-ERK. Compared with the control group, the protein level of p-JNK was 0.89-fold higher in the 5-µM Tan I-treated CML cells, while when the concentration of Tan I was increased to 10 µM, the protein level of p-JNK reached 2.09-fold higher levels than that of the control group (Figure 3B). Furthermore, Tan I also showed an inhibitory effect on protein levels of p-ERK and p-ATF-2. After the treatment with 5 µM Tan I for 48 h, the phosphorylation of ERK and ATF-2 was reduced to 52 and 67%, respectively (Figure 3A and B). Tan I treatment significantly increased the ratio of p-JNK/JNK and dramatically down-regulated p-ERK/ERK ratio. After treatment with 5 and 10 µM Tan I, the relative protein ratios of p-JNK/JNK were 0.99- and 1.41-fold higher than that of the control group, respectively (Figure 3C). However, the p-ERK/ERK ratio was reduced by 0.45- and 0.18-fold after exposure to 5 µM and 10 µM Tan I, respectively (Figure 3C). In addition, high levels of Tan I treatment increased mRNA expressions of JNK1/2 and ERK1/2 (Figure 4). According to quantitative PCR analysis, the relative mRNA expression levels of JNK1, JNK2, ERK1 and EKR2 increased by 2.25, 2.39, 1.78, and 1.24 times after treatment with 10 µM Tan I, respectively.

**Figure 5:**
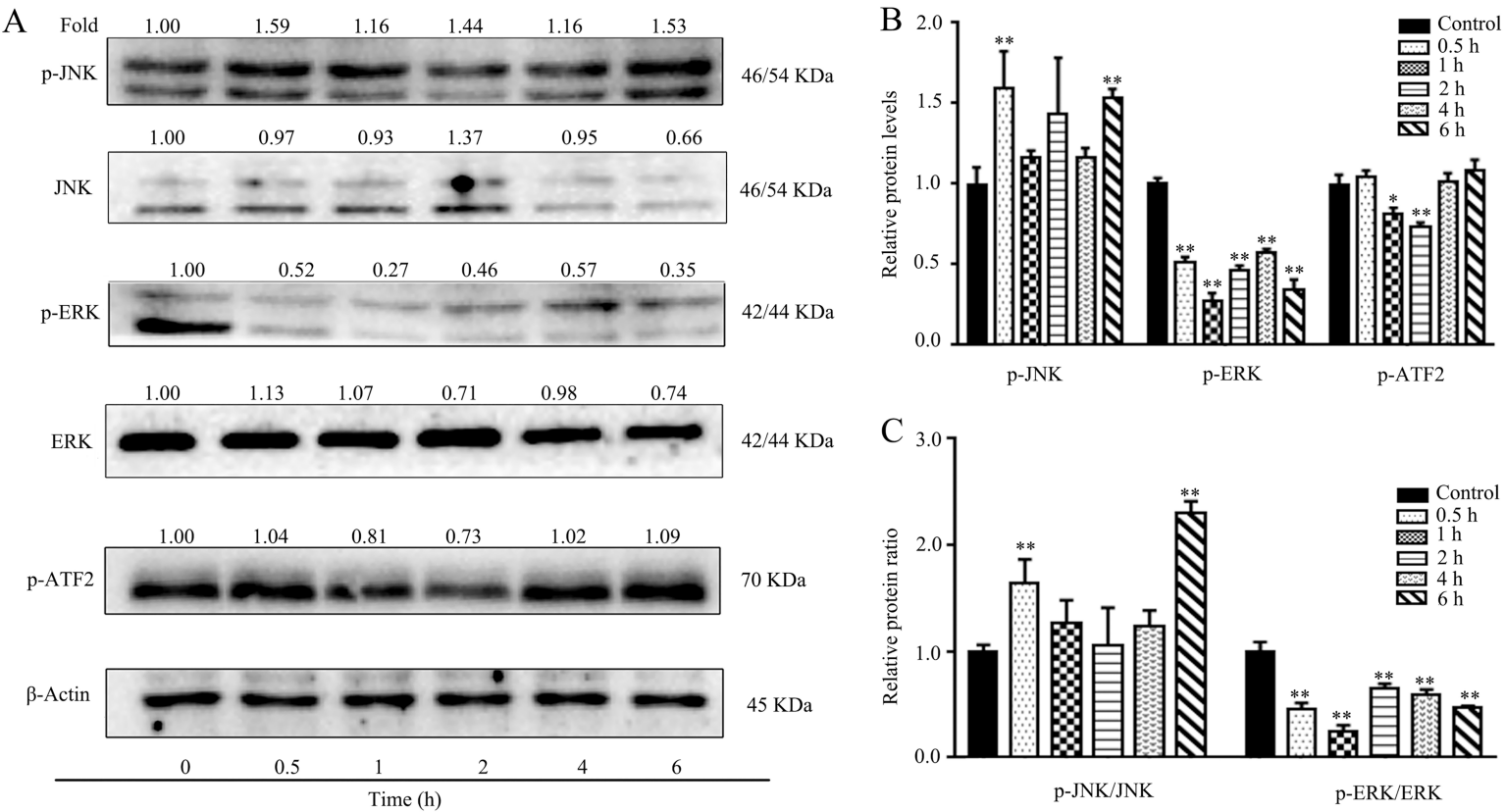
Effect of Tan I on JNK and ERK signaling pathways in K562 cells at different treatment times. **A**, Related protein expression was measured using western blotting. **B**, The relative protein levels of p-JNK, p-ERK, and p-ATF2 were quantified using Image Lab software. **C**, The relative protein level ratios of p-JNK/JNK and p-ERK/ERK. Data are reported as means±SD. *P<0.05 and **P<0.01 compared with the corresponding control group (ANOVA).

**Figure 6:**
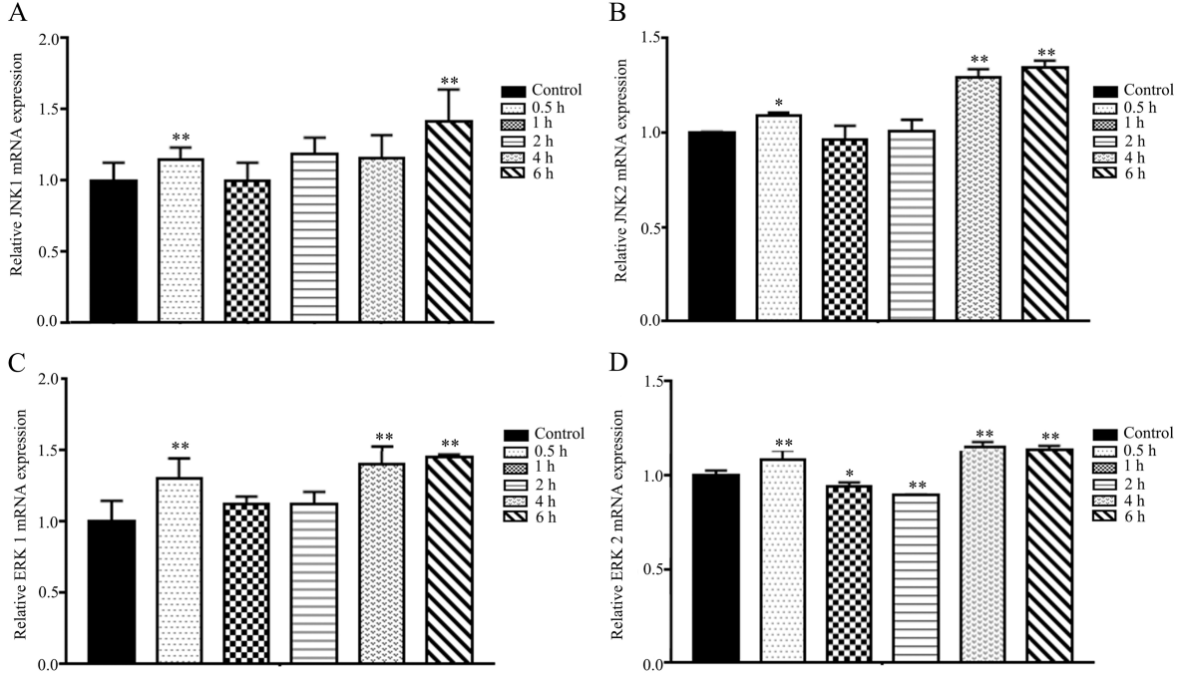
Effect of Tan I on the mRNA expression levels of JNK1/2 and ERK1/2 at different treatment times. **A**-**D**, The mRNA expression changes of JNK1/2 and ERK1/2 were determined by quantitative PCR analysis after exposure to Tan I. Data are reported as means±SD. *P<0.05, **P<0.01 compared with the corresponding control group (ANOVA).

In order to further clarify the role of Tan I in K562 cells, the phosphorylation levels of JNK, ATF-2, and ERK signaling pathways were determined at different times after treatment with Tan I. As shown in Figure 5A and B, 5 µM Tan I triggered a rapid phosphorylation level of JNK, as well as induced a quick inhibition of the phosphorylation level of ERK in K562 cells. After exposure to Tan I, the phosphorylation of JNK protein increased by 0.59 times within 30 min. Tan I significantly inhibited the phosphorylation level of ERK within a very short time. The phosphorylation of ATF-2 protein exhibited no changes within the first 6 h, suggesting an indirect regulatory mechanism of Tan I. In addition, Tan I significantly promoted the ratio of p-JNK/JNK and suppressed the p-ERK/ERK ratio, respectively (Figure 5C). The p-JNK/JNK protein ratio reached the maximum at 6 h, which was 1.32 times higher than the control group. However, the relative p-ERK/ERK expression levels dramatically decreased by 46.02 and 25.23% after Tan I treatment for 0.5 and 1 h, respectively (Figure 5A). Furthermore, the quantitative PCR results indicated that Tan I could rapidly increase the mRNA expression levels of JNK1/2 and ERK1/2 at the concentration of 5 µM in K562 cells (Figure 6). Overall, our data demonstrated that Tan I played a role in the anti-leukemia effect through JNK and ERK pathways.

## Discussion

This study investigated the anti-leukemia effect and mechanism of Tan I, and the results showed that Tan I could significantly reduce the cell viability and cause apoptosis of K562 cells. Experimental investigations indicated that Tan I showed anti-leukemia activity through the activation of cleavages of caspase proteins and PUMA, dependent on the regulation of the JNK and ERK signaling pathways. Therefore, these results may highlight the potential of Tan I in leukemia treatment by regulating cell viability and apoptosis.

Traditional Chinese medicine offers a promising treatment for CML patients with imatinib resistance, which could interact with multiple targets with fewer adverse effects and lower toxicity. For instance, andrographolide as well as its derivative NCTU-322 could induce cell differentiation, mitotic arrest, and apoptosis in imatinib-resistant CML cells via inhibiting the Bcr-Abl oncoprotein, suggesting potential therapeutic development of the two active agents against imatinib-resistant CML patients (17). Furthermore, a clinical trial demonstrated that the combination of compound Zhebei granules CZBG and chemotherapy could significantly improve the clinical remission rate of refractory acute leukemia (18). Herbal medicines are becoming a major source of anti-cancer drugs. Specifically, the famous Chinese herbal medicine *Salvia miltiorrhiza* is a rich source of active agents against tumor progression. Tanshinone IIA induced mitochondria-dependent apoptosis in KBM-5 chronic myeloid leukemia cells through targeting JNK signaling pathway. Our previous results demonstrated the synergistic effects between cryptotanshinone and imatinib on apoptosis of imatinib-resistant CML cells *in vitro*, providing a new therapeutic strategy for CML-resistant patients (19). In addition, Liu et al. (20) reported that Tan I induced CML cell apoptosis involving the inhibition of PI3k/Akt signaling pathway. In our study, Tan I significantly inhibited K562 cell growth and induced apoptosis, which was consistent with the previous reports. Here, we further demonstrated that Tan I exhibited its anti-leukemia effect in K562 cells by upregulating the activity of JNK and downregulating the activity of ERK.

The activity of ERK signaling pathway was closely related to cell proliferation, differentiation, and apoptosis in cancer cells (21). The mammalian ERK1/2 module is an evolutionarily conserved protein that could be preferentially activated in response to growth factors and phorbol esters. The aberrant activation of ERK pathway is fundamental for the occurrence and progression of various cancers (22). Therefore, specific inhibitors targeting ERK signaling pathway represent potential and attractive active agents for cancer therapy. ERK inhibitor U1206 abolished the promoting effect of SULF2 on the growth of cervical cancer cells (23). In addition, the combination of ERK inhibitor U0126 and CYP inhibitor induced a much higher inhibitory effect on ERK activity in colon cancer cells with BRAF mutant (24). However, the feedback loop control in the ERK cascade plays multiple roles in maintaining cellular homeostasis and sensitivity in cancer cells (25). The PI3K/mTOR kinase inhibitor BEZ235 led to a rapid feedback activation of p-ERK in pancreatic cancer cells (26). The Src-dependent ERK reactivation in PC9-GR was closely related to gefitinib resistance (27). The active TCM agent icaritin even triggered sustained activation of ERK1/2 and induced apoptosis in human endometrial cancer cells, suggesting more complex regulation mechanisms of ERK pathway (28). ERK1/2 also locates in the critical position in regulating cell apoptosis and chemo-sensitivity of CML. Previous reports indicated that enhanced activity of ERK pathway was accompanied by imatinib resistance in CML cells (29). Imatinib resistance could be reversed by targeting the elevated activity of Lyn/ERK signaling pathway in K562R leukemia cells (30). The ERK pathway has become a promising target to induce cell apoptosis and restore imatinib sensitivity of CML cells.

The combination of hydroxychavicol and glutathione synthesis inhibitor buthionine sulfoximine was demonstrated to induce a synergistic apoptosis effect in CML cells via increasing the phosphorylation of ERK1/2 (31). On the other hand, many active agents exhibited anti-leukemia activity by inhibiting the phosphorylation of ERK1/2 in CML cells. Matrine significantly reduced the expression level of phospho-ERK1/2, leading to apoptosis in K562 and HL-60 cells (32). Bruceine D caused a proliferative inhibitory effect through inhibiting the phosphorylation of ERK and Akt in K562 cells (33). Our results also demonstrated that Tan I significantly inhibited the p-ERK protein expression level in K562 cells. In addition, Tan I exhibited positive regulation on the mRNA expression levels of ERK1 and ERK2 (Figures 4 and 6). Moreover, Tan I triggered the inhibition of ERK signaling pathway within 30 min, suggesting Tan I down-regulated the phosphorylation level of ERK directly.

Activating transcription factor 2 (ATF2) is a multifunctional transcription factor, which plays a crucial role in oncogenic transformation and tumorigenesis (34). In cervical cancer cells, miR-204 inhibited cell proliferation and induced apoptosis via downregulating the expression of ATF2 (35). Furthermore, ATF2 siRNA inhibited growth and promoted apoptosis in PCa cell lines, which led to increased gemcitabine sensitivity (36). These results showed that ATF2 could be used as a promising target for cancer therapy. However, the ATF2 signaling pathway exhibited multiple roles involving a complex regulatory mechanism. For example, the inhibition activity of ATF2 was associated with NB4 cells apoptosis and cycle arrest when treated with selenite (37). In addition, JNK suppresses tumor formation via a gene-expression program mediated by ATF2 (38). The microtubule disrupting agent BNC105 could lead to rapid apoptosis and result in activation of JNK/ATF2 pathway in chronic lymphocytic leukemia cells (39). Furthermore, the MAPK regulatory mechanism and the expression and activity of ATF2 were also closely related to certain mechanisms regarding TNF-α and miR-299-5p regulation (40). Our data confirmed that Tan I inhibited the expression of p-ATF2 and up-regulated the expression of p-JNK in CML cells (Figure 3A and B). These results suggested that Tan I regulated the activity of ATF2 independent of JNK regulatory pathway, indicating the involvement of other regulation mechanisms. Moreover, Tan I treatment led to almost no changes in the phosphorylation level of ATF2 during the first 6 h, further confirming an indirect regulation on ATF2 activity (Figure 5A and B).

In conclusion, our study demonstrated that Tan I could inhibit growth and cause apoptosis accompanied by activation of JNK as well as inhibition of ERK and ATF2 signaling pathways in K562 cells. Tan I treatment significantly induced higher apoptotic cell fractions of CML cells via activating the cleavages of caspase proteins and increasing the expression of PUMA. These results may provide important insights for the potential discovery and development of Tan I as a novel therapeutic drug for chronic myeloid leukemia.
